# Physicochemical and Functional Properties of Type B Gelatin Obtained from Nile Tilapia (*Oreochromis niloticus*) Scales Using Hydro-Extraction: Effect of Ultrasound Pretreatment

**DOI:** 10.3390/pharmaceutics18040463

**Published:** 2026-04-09

**Authors:** Kelly Triana-Jiménez, Carlos Alonso, Milena A. Vega, Pablo Juanes-Velasco, Iván Menéses-Rivera, Mario Velásquez-Lozano

**Affiliations:** 1 Departamento de Ingeniería Química y Ambiental, Universidad Nacional de Colombia, Bogotá AK 30 45-03, Colombia; imenesesr@unal.edu.co (I.M.-R.); mevelasquezl@unal.edu.co (M.V.-L.); 2 Departamento de Ingeniería Química y Textil, Facultad de Ciencias Químicas, Universidad de Salamanca, 37008 Salamanca, Spain; c.alonso@usal.es; 3 Instituto de Investigación Biomédica de Salamanca (IBSAL), Complejo Asistencial de Salamanca, Paseo de San Vicente, 58, 37008 Salamanca, Spain; 4 Translational and Clinical Reserach Program, Cancer Research Center (IBMCC, CSIC-University of Salamanca), Cytometry Service, NUCLEUS, Departamento de Medicina, Universidad de Salamanca, 37008 Salamanca, Spain; pablojuanesvelasco@usal.es; 5 Biomedical Research Networking Centre Consortium of Oncology (CIBERONC), Instituto de Salud Carlos III, 28029 Madrid, Spain; 6 Plataforma de Proteómica Clínica, Instituto de Investigación Biomédica de Salamanca (IBSAL), 37007 Salamanca, Spain

**Keywords:** fish gelatin, collagen, Nile tilapia (*Oreochromis niloticus*), ultrasound-assisted extraction, protein

## Abstract

**Background:** In this study, type B gelatin was extracted from *Oreochromis niloticus* scales under hydrothermal conditions at 60 °C to evaluate the effect of ultrasound-assisted pretreatment on its structural, physicochemical, thermal, and functional properties. **Methods:** Gelatin obtained with and without ultrasound pretreatment was systematically characterized through molecular weight analysis, proteomic profiling, size determination, surface morphology, proximate composition, thermal behavior, and gelation-related functional properties in order to assess the influence of the extraction method on gelation performance. **Results:** Ultrasound pretreatment slightly increased gelatin yield from 1.46 to 1.70%, indicating enhanced collagen solubilization. Proteomic analysis confirmed the predominance of fibrillar collagen proteins in both samples, although differences in protein distribution were observed. Furthermore, weight-average molecular weight analysis revealed a reduction from 212.3 ± 11.8 to 170.9 ± 13.2 kDa in the ultrasound-treated sample, suggesting partial fragmentation of collagen chains induced by cavitation effects. Structural modifications were also reflected in increased porosity and surface changes, contributing to improved colloidal stability. However, these changes significantly affect the functional behavior of the gelatin. Ultrasound-treated sample exhibited limited gel-forming capacity and failed to form stable gels at the evaluated concentration, despite complete dissolution. In contrast, gelatin extracted without ultrasound treatment retained higher-molecular-weight fractions and formed stable gels at both 5 and 10% (*w*/*w*). Thermal and spectroscopic analyses suggested that the fundamental collagen structure was preserved in both samples, although differences were observed in thermal degradation behavior. **Conclusions:** These results highlight the importance of controlling ultrasound-assisted extraction conditions to balance collagen recovery with the preservation of molecular integrity required for gelation, providing insights for the development of sustainable fish-derived biomaterials for pharmaceuticals and biomedical applications.

## 1. Introduction

Collagen is a major structural protein of the extracellular matrix and is widely distributed in connective tissues such as skin, bones, tendons, cartilage, and internal organs. It is one of the most abundant proteins in vertebrates, accounting for approximately 25% of the total protein content, and plays an instrumental role in the mechanical strength, flexibility, and structural integrity of biological tissues [1]. To date, more than 27 different types of collagen have been identified and are generally classified as fibrillar or non-fibrillar collagens according to their molecular structure and biological function [2]. Among them, type I collagen is the most abundant and represents the principal collagen component in skin, bones, tendons, and fish scales, making it particularly relevant for industrial and biomedical applications [3].

From a molecular perspective, collagen is characterized by a triple-helix conformation built by three polypeptide chains rich in glycine, proline, and hydroxyproline, arranged in a repetitive Gly-X-Y sequence [4]. This molecular organization allows for extensive intermolecular interactions, primarily through hydrogen bonding, which confer high mechanical strength and structural stability to collagen. Gelatin, in turn, is obtained through the partial hydrolysis and thermal denaturation of collagen, processes that disrupt the triple-helix structure and generate polypeptide chains of lower molecular weight, typically ranging from 15 to 250 kDa [5,6]. Higher-molecular-weight fractions retain gel-forming capacity, whereas lower-molecular-weight fractions are commonly referred to as gelatin hydrolysates and lack gelling capacity [7].

Due to its excellent biocompatibility, biodegradability, and low immunogenicity, gelatin has gained considerable interest in pharmaceutical and biomedical applications. Gelatin-based materials are widely used in the formulation of hard and soft capsules, drug delivery systems, wound healing materials, injectable hydrogels, and scaffolds for tissue engineering, where their physicochemical and structural properties influence drug stability, release kinetics, and mechanical performance [8,9,10,11]. Consequently, controlling parameters such as molecular weight distribution, gel strength, and thermal stability is essential, as these properties directly influence drug stability, release kinetics, and the mechanical performance of gelatin-based pharmaceutical formulations.

Traditionally, gelatin has been produced from bovine and porcine skins and bones. However, these sources present limitations related to religious restrictions, concerns about zoonotic disease transmission, and environmental issues associated with livestock production [12]. In this context, fish-derived gelatin has emerged as a promising alternative due to its high biocompatibility, reduced risk of disease transmission, and the possibility of valorizing fish-processing byproducts within a circular bioeconomy framework [13,14,15]. Among these byproducts, fish scales are particularly attractive because of their high collagen content and relatively low lipid levels, which facilitate the collagen extraction and purification process [16,17].

The increasing interest in marine-derived biomaterials is also reflected in the global market trends for fish gelatin, which is expected to experience sustained growth driven by the increasing demand for pharmaceutical, nutraceutical, and biomedical products derived from sustainable sources. In 2025, the global fish gelatin market was estimated at approximately USD 305.1 million and is projected to reach around USD 414 million by 2034 [18].

Gelatin extraction typically involves acid or alkaline pretreatment steps designed to weaken the collagen structure through the disruption of hydrogen, electrostatic, and covalent bonds [19]. Depending on the extraction conditions, gelatin can be classified as type A or type B, each exhibiting different isoelectric points and functional properties. Consequently, the conversion of collagen into gelatin is strongly influenced by parameters such as pH, temperature, and extraction time, which determine the molecular weight distribution and, therefore, the physicochemical properties of the resulting biomaterial [2,20]. Fish-derived gelatin often exhibits lower thermal stability than mammalian obtained; however, this limitation can be mitigated through the optimization of extraction conditions and processing technologies [2,20,21].

In recent years, innovative extraction technologies have been developed to improve yield and functional properties while reducing processing time, energy consumption, and chemical usage. Among these techniques, ultrasound-assisted extraction has attracted increasing attention as a green processing technology, capable of enhancing mass transfer through acoustic cavitation phenomena [22,23,24]. The collapse of cavitation bubbles generates localized shear forces which can disrupt collagen fibrils, improve solvent penetration, and facilitate collagen solubilization. As a result, ultrasound treatment may modify the molecular structure, molecular weight distribution, and functional properties [5,8,25,26]. Recent studies have highlighted the potential of ultrasound technology to influence the structure–function relationships of these proteins, affecting protein conformation, intermolecular interactions, and physicochemical behavior of the resulting biomaterials [27].

However, despite these advances, studies addressing the influence of ultrasound pretreatment on gelatin extracted from freshwater fish scales remain limited, particularly regarding the combined evaluation of physicochemical, thermal, and proteomic characteristics [28]. Understanding how ultrasound processing affects collagen structure and gelatin functionality is essential for the development of high-value biomaterials derived from fish-processing byproducts [27]. This approach provides new insight into how ultrasound-assisted extraction influences the structural and functional properties of gelatin obtained from freshwater fish scales, which remains an underexplored raw material for pharmaceutical and biomedical biomaterials.

**Figure 1:**
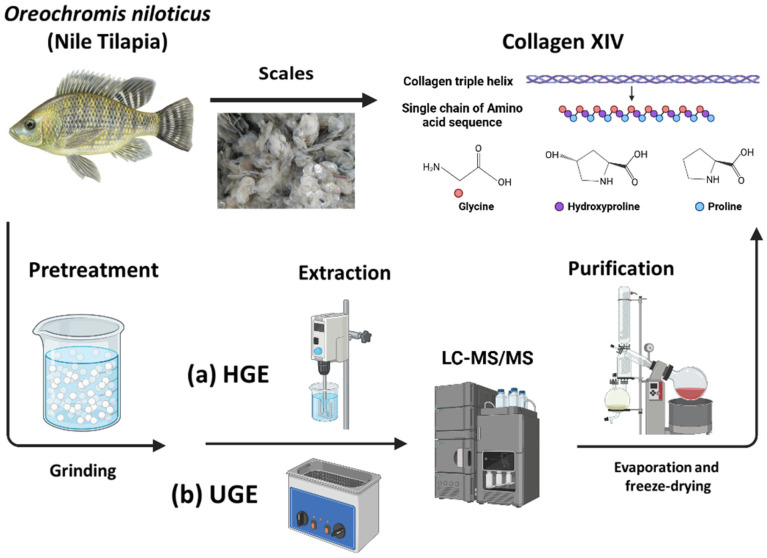
Schematic overview of gelatin extraction from *Oreochromis niloticus* scales. The process includes scale grinding, alkaline pretreatment to weaken the collagen structure by hydro-extraction (HGE) or ultrasound-assisted extraction (UGE), analysis by liquid chromatography–tandem mass spectrometry (LC-MS/MS), and purification by evaporation and freeze-drying. Created with BioRender.com.

Therefore, the aim of this study is to investigate the physicochemical, thermal, and proteomic properties of gelatin extracted from *Oreochromis niloticus* scales under hydrothermal conditions at 60 °C, evaluating the effect of ultrasound-assisted pretreatment on its structural and functional characteristics. Gelatins obtained with and without ultrasound, classified as type B due to alkaline pretreatment, were analyzed to determine how the extraction method influences molecular weight distribution, thermal stability, and protein composition. Particular attention was given to identifying structural factors that could enhance the suitability of fish-derived gelatin for future biomedical and pharmaceutical scaffold applications (Figure 1).

## 2. Materials and Methods

### 2.1. Fish Scales Preparation

Fish scales (FSs) of Nile tilapia (*Oreochromis niloticus*) were obtained from a fish-processing plant (provided by Asesoría y Acuicultura ASYA, Villavicencio, Colombia) stored on ice, and immediately transported to the laboratory. Upon arrival, the FSs were packed in polyethylene bags and stored at −20 °C until use. To remove non-collagenous organic matter, the FSs were soaked in 0.1 M NaOH (Merck KGaA, Darmstadt, Germany) at room temperature for 24 h using a scale-to-solution ratio of 1:5 (*w*/*w*). After alkaline treatment, the solution was removed by filtration, and the scales were washed several times with distilled water (DW) under agitation for around 30 min per washing cycle. This procedure was repeated until the pH of the washing water reached approximately 7.4, indicating the removal of residual alkali. Subsequently, hydro-extraction was carried out with DW at a ratio of 1:4 (FS: DW) at 60 °C for 3 h to obtain fish gelatin. The moisture content of the raw material was determined by drying the sample in a muffle furnace at 105 °C until constant weight was reached (approximately 4 h). According to supplier information, this fish-processing plant also processes red tilapia (*Oreochromis* sp.) and cachama (*Colossoma* sp.).

### 2.2. Gelatin Extraction

Gelatin extraction was carried out using a hydro-extraction process with distilled water (DW). A mixture of FSs and DW was prepared at a 1:4 ratio (g of FS per g of DW) and placed in a 2 L beaker on a heating plate. The mixture was maintained at 60 °C for 3 h under constant stirring at 240 rpm.

In addition to the conventional hydro-extraction process (sample denoted as HGE), the effect of ultrasound pretreatment prior to hydro-extraction was evaluated (sample denoted as UGE). For this purpose, the FS suspension was subjected to sonication in an ultrasonic bath model WUC-D06H (Daihan Scientific, Wonju, Republic of Korea) at 30 °C for 30 min using a frequency of 40 kHz and a nominal ultrasonic power of 230 W in continuous mode. During sonication, the sample was maintained under constant stirring (240 rpm) to ensure homogeneous exposure before proceeding to the hydro-extraction step.

Subsequently, the samples were filtered at room temperature (RT). The resulting liquid fraction was collected in 50 mL Falcon tubes and centrifuged at 10,000 rpm and 10 °C to remove dark-colored insoluble impurities, including melanin present in FSs [29]. During this step, phase separation was assessed by varying the centrifugation time in 10 min intervals, and 30 min was determined to be the optimal time to achieve effective clarification of the sample. No additional purification steps, such as ultrafiltration, were performed to maintain comparable extraction conditions for both gelatin samples. After centrifugation, the supernatant was concentrated by rotary evaporation at 50 °C and 65 rpm, once the system temperature had stabilized (Ika, Staufen, Germany). Subsequently, the concentrated samples were frozen at −20 °C and then lyophilized for 48 h at −105 °C (Labconco, Kansas City, MO, USA). Following this stage, the gelatin samples were ready for further analysis and characterization.

#### Yield

To determine gelatin extraction yield, Equation (1) was used, where mGE (liofilized) corresponds to the mass of lyophilized gelatin and mScales (b.s) to the mass of the FSs prior to the 0.1 M NaOH pretreatment, expressed on a dry basis. For this purpose, fresh scale samples (2 g) were placed in ceramic crucibles and dried at 105 °C for 4 h, followed by calcination at 550 °C to determine moisture content, volatile solids, and ash content. All measurements were performed in triplicate, in accordance with NREL/TP-510-42621:2008 standard [30].

(1) $$Yield\;\left(\%\right)=\frac{mGE\;\left(liofilized\right)}{mScales\;\left(b.s\right)}\times 100\%$$

### 2.3. Physicochemical Characterization: Hydrodynamic Diameter, Zeta Potential, Weight-Average Molecular Weight, Isoelectric Point, and Superficial Structure

The hydrodynamic diameter of lyophilized gelatin samples (HGE and UGE) was determined with dynamic light scattering (DLS) using a Malvern Zetasizer Nano ZS (Malvern Panalytical Ltd., Malvern, UK). For this purpose, lyophilized gelatin solutions were prepared in DW at a concentration of 0.1% (*w*/*w*). The solutions were preheated to 20 and 50 °C under magnetic stirring at 360 rpm for 1 h to promote complete dissolution and sample homogeneity. Prior to the measurements, the samples were filtered through cellulose quantitative grade 42 Whatman^®^ filters with a pore size of 2.5 µm (Cytiva, Uppsala, Sweden) to remove possible aggregates or undissolved particles and subsequently sonicated for 20 s to ensure proper dispersion. Additionally, control experiments were conducted on HGE samples to evaluate the effect of the dispersion method, in which vortex mixing at 20 s and 1000 rpm was applied instead of sonication prior to DLS measurements. Hydrodynamic diameter measurements were performed at pH 6.8. In addition, the surface charge of the gelatin samples was evaluated after dispersion at three different pH values (6.8, 7.6, and 10.7), using TRIS [(hydroxymethyl)aminomethane] buffer adjusted to the corresponding pH. All measurements were carried out at 25 °C using non-invasive backscatter (NIBS) detection with a scattering angle of 175°, corresponding to the standard configuration of the instrument. The polydispersity index (PDI) of the samples was obtained from the DLS measurements and automatically calculated by the instrument software using the cumulant analysis of the intensity autocorrelation function. Each measurement was performed in triplicate.

The weight-average molecular weight (Mw) of the two gelatin samples was determined with static light scattering (SLS) using the same instrument and applying the protein size analysis function. For this purpose, solutions of both gelatin samples were prepared in PBS buffer (pH 7.4) over a concentration range of 0.01 to 0.1 g/mL. Toluene was used as a Rayleigh ratio standard for calibration (Figure S1). Based on the obtained measurements, Rayleigh plots were constructed in which the intercept corresponds to the inverse of the weight-average molecular weight (1/Mw), allowing the Mw to be estimated for each sample [31].

The isoelectric point (pI) of the gelatins was determined using potentiometric titration. For this purpose, NaOH (0.1 M) and HCl (0.25 M) solutions were used (Merck KGaA, Darmstadt, Germany). Gelatin samples were prepared at concentrations of 0.22 mM for HGE and 0.23 mM for UGE in the acidic solution. The samples were maintained under constant stirring while the pH was continuously monitored. Subsequently, the NaOH solution was gradually added from a burette in a controlled manner, recording the variation in pH as a function of the added volume. The pI was determined from the titration curve by calculating the inflection point using the second derivative of pH with respect to the volume of base added (Figure S2).

Finally, the microstructure of the two gelatin samples was evaluated by scanning electron microscopy (SEM) using an FESEM Ultra Plus instrument (Carl Zeiss Microscopy GmbH, Jena, Germany). For this analysis, the gelatin samples were first lyophilized and then transversely sectioned to expose their internal structure. The resulting gelatin sections were subsequently placed onto a silicon nitride substrate. To facilitate adhesion, a drop of DW was previously deposited onto the substrate, and the samples were allowed to dry for 24 h. Prior to image acquisition, the samples were coated by sputter deposition with a thin iridium layer (5 nm) to minimize surface charging effects. The micrographs were acquired at an accelerating voltage of 3 kV using a secondary electron detector (SE/in-lens).

### 2.4. Proximate Composition: Moisture, Ash, and Total Fat

Proximate composition (moisture, ash, and total fat) of the two gelatin samples was determined according to AOAC standard methods (AOAC, 1990). First, the moisture content was determined gravimetrically by drying approximately 2 g of fish gelatin sample in a pre-dried porcelain crucible at 105 °C until constant weight was reached (approximately 4 h). After drying, the samples were cooled to room temperature in a desiccator and weighed. The moisture content was calculated as the percentage of weight loss relative to the initial sample mass.

Subsequently, ash content was determined using the dry ashing method. Approximately 2 g of sample was weighed into a previously ignited and tared porcelain crucible, pre-ashed over a Bunsen burner, and then incinerated in a muffle furnace (Terrigeno S.A.S, Medellín, Colombia) at 550 °C for approximately 8 h until a white ash was obtained, indicating the complete removal of organic matter. After cooling in a desiccator, the crucibles were weighed, and the ash content was calculated on a dry weight basis.

Finally, the total fat content was determined gravimetrically through solvent extraction. Approximately 2 g of sample were placed in a cellulose extraction thimble and extracted with diethyl ether for about 8 h. After the extraction process, the solvent was removed using a rotary evaporator (Ika, Staufen, Germany), and the remaining lipid residue was dried at 100 °C for 60 min before weighing. Fat content was expressed as a percentage of the initial sample mass. All measurements were performed in triplicate. Statistical analysis was carried out using student’s independent *t*-tests for equal or unequal variances, as appropriate, with a level of significance *p* < 0.05.

### 2.5. Fourier Transform Infrared Characterization

The chemical structure of the two gelatin samples was analyzed with Fourier transform infrared (FTIR) spectroscopy using a Spectrum Two™ spectrometer (PerkinElmer, Waltham, MA, USA). Spectra were recorded over the spectral range of 4000 to 800 cm^−1^ with a resolution of 1 cm^−1^, collecting 24 scans per sample. Prior to analysis, the lyophilized samples were ground and dried at 37 °C for 3 days to remove any residual moisture; subsequently, KBr pellets were prepared and subjected to spectroscopic analysis.

### 2.6. Thermogravimetric Analysis

Thermogravimetric analysis (TGA) was performed to evaluate the thermal stability of the gelatin samples using a Mettler Toledo TGA/DSC 1 STAR instrument (Mettler-Toledo International Inc., Greifensee, Switzerland). Prior to analysis, the lyophilized samples were conditioned in a desiccator for 18 h at 40 °C to minimize moisture interference. Approximately 2.9 mg of HGE and 7.6 mg of UGE were weighed in aluminum crucibles with a capacity of 70 µL, while an empty crucible was used as reference. Measurements were carried out under a nitrogen atmosphere at a constant flow rate of 40 mL/min, applying a heating ramp of 10 °C/min over a temperature range of 25 to 900 °C. The instrument was calibrated prior to the measurements according to the standard procedures recommended by the manufacturer.

### 2.7. Differential Scanning Calorimetry (DSC)

In addition, differential scanning calorimetry (DSC) tests were performed using a high-pressure calorimeter HP DSC 1 (Mettler-Toledo International Inc., Greifensee, Switzerland) under a nitrogen atmosphere, with a constant flow rate of 50 mL/min and a heating rate of 10 °C/min, over a temperature range from 30 to 400 °C. Prior to the analysis, the lyophilized samples were kept in a desiccator for 18 h.

### 2.8. Gel-Forming Capacity and Water Holding Capacity (WHC)

To evaluate gel-forming capacity, solutions of laboratory-grade bovine gelatin (PN 44045, BDH Chemicals Ltd., Poole, UK) and the two gelatin samples were prepared in DW at 70 °C at concentrations of 5 and 10% (*w*/*w*) using 10 mL test tubes. This temperature was selected to facilitate the complete dissolution of gelatin in DW and ensure proper protein hydration prior to gel formation. The samples were homogenized by vigorous mixing and subsequently cooled at 4 °C for 1 h. After the cooling period, the samples were removed from refrigeration and visually assessed for gel formation. All determinations were performed in triplicate and quantified by gravimetric measurements.

Additionally, water holding capacity (WHC) was determined by placing 1 g of sample in a 50 mL Falcon tube and adding 30 mL of DW at room temperature. The mixture was thoroughly homogenized to ensure uniform dispersion. To allow swelling of the protein matrix, the suspension was allowed to stand for 30 min at room temperature. The tubes were then centrifuged at 4000 rpm for 20 min at 10 °C. After centrifugation, the supernatant was carefully discarded, and the mass of the hydrated gel remaining in the tube was recorded. All measurements were performed in triplicate. Finally, WHC was calculated as the amount of water retained per gram of dry sample using Equation (2), where $W_{w}$ is the weight of the hydrated gel after centrifugation and $W_{d}$ is the initial weight of the dry sample [32].

(2) $$WHC\;\left(\%\right)=\frac{W_{w}-W_{d}}{W_{d}}\times 100\%$$

### 2.9. Protein Content and Peptide Characterization

#### 2.9.1. Protein Content

Protein content was quantified using a modified Bradford method. For the construction of the calibration curve, standard solutions of laboratory-grade bovine gelatin (PN 44045, BDH Chemicals Ltd., Poole, UK) were prepared over a concentration range of 30 to 300 μg/mL using 0.25 M acetic acid as the solvent (Merck KGaA, Darmstadt, Germany). For the analysis of the gelatin samples, solutions with an approximate concentration of 120 μg/mL were prepared, maintaining the same solvent concentration. The Bradford assay was performed using a Quick Start™ Bradford Protein Assay 1× Bio-Rad reagent (Bio-Rad Laboratories, Inc., Hercules, CA, USA). Prior to measurement, proteins were denatured with the addition of sodium dodecyl sulfate (SDS, Sigma-Aldrich, St. Louis, MO, USA). Assay samples were prepared by mixing 2.5 mL of Bradford reagent, 50 μL of SDS (0.0035%), and 50 μL of the sample dissolved in acetic acid. Absorbance was measured at 595 nm using a spectrophotometer. All determinations were performed in triplicate.

#### 2.9.2. Sample Preparation for LC-MS/MS Analysis

For the preparation of gelatin samples (HGE and UGE) for mass spectrometry (MS) analysis, the iST (in-StageTip) method was employed using the iST sample preparation kit for cell pellets and precipitated proteins (PreOmics GmbH, Planegg, Germany), strictly following the manufacturer’s protocol.

Lysis: Each sample was treated with 50 µL of lysis buffer (per 1–100 µg of protein) and incubated in a thermomixer with agitation for 10 min at 1000 rpm and 95 °C. During this step, protein lysis, denaturation, reduction, and alkylation were carried out simultaneously.

Enzymatic digestion: Subsequently, 50 µL of digestion buffer containing trypsin and Lys-C were added, and the samples were incubated for 1 h and 30 min at 37 °C and 500 rpm. After digestion, 100 µL of enzyme reaction stop buffer were added, followed by incubation for 1 min at 500 rpm and RT.

Purification: Peptide purification was performed using a C18 filter. The digested peptide solution was loaded onto the filter and centrifuged for 3 min at 3800 rpm. Two washing steps were then carried out: First, 200 µL of organic wash buffer No. 1 were added to remove hydrophobic residues, followed by centrifugation for 3 min at 3800 rpm. Second, 200 µL of aqueous wash buffer No. 2 were added to remove hydrophilic residues, followed by centrifugation under the same conditions. Peptides were subsequently eluted by adding 100 µL of elution buffer and centrifuging for 3 min at 3800 rpm. This elution step was performed twice, yielding a final volume of approximately 200 µL of eluted peptides.

The eluted peptides were dried in a concentrator and, once the solvent was completely evaporated, reconstituted in the appropriate volume of loading buffer to achieve a final concentration of 1 µg/µL. The samples were mixed for 5 min at 500 rpm and RT and were then ready for MS analysis.

#### 2.9.3. LC-MS/MS Analysis

Liquid chromatography–tandem mass spectrometry (LC-MS/MS) analysis was performed using an Evosep One liquid chromatography system (Evosep Biosystems, Odense, Denmark) coupled to a timsTOF Pro 2 mass spectrometer (Bruker Corporation, Billerica, MA, USA), which enables peptide ion mobility analysis through trapped ion mobility spectrometry–time-of-flight (timsTOF). Peptide separation was achieved using a high-throughput method, allowing the analysis of 60 samples per day with an analytical column (8 cm × 100 µm; Evosep Biosystems, Odense, Denmark). Data acquisition was carried out using a data-dependent acquisition method with parallel accumulation–serial fragmentation (DDA-PASEF), over a mass-to-charge (*m*/*z*) range of 100 to 1700 and an ion mobility range of 0.60 to 1.60 1/K_0_, with a cycle time of 100 ms. Proteomic data corresponding to the characterization of both gelatin samples have been deposited in the PRIDE repository under accession number PXD071911.

### 2.10. Statistical Analysis

All experiments were performed in triplicate unless otherwise specified. Results are expressed as mean values ± standard deviation (SD). Statistical analysis was carried out using Student’s *t*-tests (paired or independent, with equal or unequal variances as appropriate for each case) to evaluate differences between samples and treatments. A level of *p* < 0.05 was considered statistically significant.

## 3. Results and Discussion

### 3.1. Gelatin Extraction: Yield

To estimate the collagen extraction yield with and without sonication pretreatment, the dry mass of the scales was used as the reference basis. The scales were found to have a moisture content of 56.71%, a volatile solids content of 27.95%, mainly associated with the protein and lipid contents, and an ash content of 15.34%. These moisture values are comparable to those reported by [33] for carp and lizardfish scales (55.67 and 64.85%, respectively). Likewise, Arellano et al. reported an ash content of 16.74% for *Oreochromis niloticus* scales, which is similar to the value obtained in the present study [34].

The sample subjected to ultrasound pretreatment (UGE) exhibited a higher yield of extracted fish gelatin (1.70%) compared to the sample without pretreatment (HGE, 1.46%). This increase can be attributed to the mechanical effect of ultrasound on the surface of the scales, which promotes tissue disruption and enhances protein migration [4,5,35]. However, these values are relatively lower than those reported in the literature (2.12 and 3.51%) for extractions conducted at 60 °C [36,37]. This behavior may be related to the extraction method employed, including alkaline pretreatment, the limited solid-to-liquid ratio, the extraction temperature, or the need for further reduction in scale particle size, all of which may restrict protein migration from within the tissue. However, despite the relatively low yield, the gelatin obtained exhibited greater firmness and suitable gelation properties over a pH range between 5 and 10, as will be discussed later [38].

### 3.2. Physicochemical Characterization: Hydrodynamic Diameter, Zeta Potential, Weight-Average Molecular Weight, Isoelectric Point, and Superficial Structure

**Figure 2:**
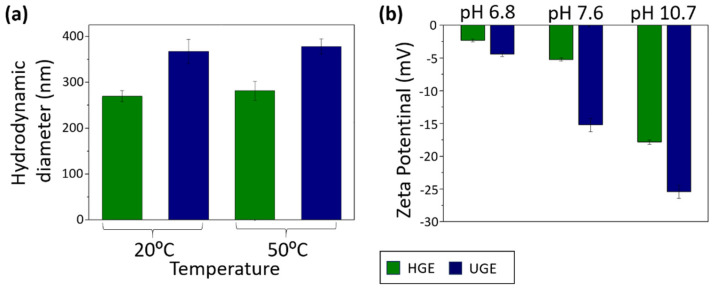
(**a**) Hydrodynamic diameter of UGE and HGE gelatin samples measured DLS at 20 and 50 °C. (**b**) Zeta potential of UGE and HGE gelatin samples measured at different pH values.

The hydrodynamic diameter of the two gelatin samples studied was analyzed using DLS (Figure 2a). According to the obtained results, the UGE gelatin exhibited a larger hydrodynamic diameter (20 °C: 367.3 ± 26.3 nm; 50 °C: 378.1 ± 17.0 nm) compared to the HGE gelatin (20 °C: 269.4 ± 12.1; 50 °C: 281.4 ± 20.4 nm), with no statistically significant differences observed between the two temperatures evaluated (*p* > 0.05). This behavior may be attributed to the presence of relatively larger aggregates in the UGE sample, which is consistent with its higher PDI values (20 °C: 0.672; 50 °C: 0.686) in comparison to those of the HGE sample (20 °C: 0.516; 50 °C: 0.554. The increased hydrodynamic diameter and hydrophobicity of UGE sample may be associated with the formation of insoluble aggregate during sonication pretreatment. Ultrasound waves generate shear forces and microjets through cavitation, which can modify the secondary and tertiary structure of proteins, leading to changes in aggregate size caused by bond disruption or exposure of hydrophobic groups, depending on processing conditions such as solvent-to-sample ratio, frequency, power, time and temperature [27,39]. Similar effects have been reported for various proteins, including those derived from fish [40,41,42]. Additionally, the relatively high PDI values indicate a highly polydisperse colloidal system, suggesting that the measured sizes correspond to heterogeneous gelatin aggregates or molecular assemblies dispersed in solution rather than discrete solid particles [43,44]. To assess whether the dispersion method applied prior to DLS measurements could influence the obtained hydrodynamic diameter, additional experiments conducted on HGE samples comparing vortex mixing and sonication. As show in Table S1, at 20 °C no statistically significant differences were observed between control samples (313.87 ± 39.10 nm), vortex-treated samples (304.70 ± 29.26 nm), and sonicated samples (269.45 ± 12.09 nm) (*p* > 0.05). At 50 °C, both treatments resulted in a decrease in hydrodynamic diameter compared to the control (338.53 ± 28.32 nm), with values of 236.68 ± 13.20 nm for vortex and 281.4 ± 20.43 nm for sonication (*p* < 0.05). However, no statistically significant differences were found between vortex and sonication (*p* > 0.05), suggesting that both dispersion methods have an equivalent effect on hydrodynamic diameter. These results indicate that the short sonication step used prior to DLS measurements does not introduce significant bias and does not interfere with the interpretation of the sonication effects discussed in this study.

The higher collagen content in the UGE sample may contribute to greater colloidal stability of the system, as evidenced by the more negative zeta potential values compared to the HGE sample (Figure 2b). Furthermore, for both gelatin samples, increasing the measurement pH resulted in more negative zeta potential values, thereby enhancing the colloidal stability of collagen at higher pH levels.

In addition, the zeta potential results reveal differences in the colloidal stability of the gelatin samples, in agreement with the hydrodynamic diameter and PDI results (Figure 2b). The UGE sample exhibited more negative zeta potential values than the HGE sample, which may be attributed to its higher collagen content and the sonication pretreatment, the consequent exposure of ionizable groups and increasing of the surface charge density. Furthermore, for both gelatin samples, higher pH led to progressively more negative zeta potential values, intensifying electrostatic repulsion between aggregates, and contributing to enhanced colloidal stability of the gelatin samples [45].

Moreover, the Mw values of collagen in the HGE and UGE samples were 212.3 ± 11.8 kDa and 170.9 ± 13.2 kDa, respectively. Accordingly, the HGE sample exhibited an Mw approximately 24% higher than that of the sample subjected to ultrasound pretreatment, suggesting a higher content of β chains. These results are consistent with values reported in the literature, considering the effect of cavitation on protein structure during ultrasound pretreatment [46].

Furthermore, to confirm the type of gelatin obtained (A or B), the pI was determined, as this parameter is directly related to the net charge of the protein. In general, type A gelatin, produced through acid treatment, typically exhibits pI values between 7 and 9, whereas type B gelatin, obtained through alkaline pretreatment, usually presents pI values between 4 and 5 [47,48,49].

In this study, the pI values obtained were 4.41 for HGE gelatin and 4.90 for UGE gelatin, indicating that both samples exhibited a slightly acidic character. Moreover, these values fall within the typical range reported for type B gelatin, suggesting that the extracted gelatins corresponded to this type. This acidic behavior is commonly associated with alkaline processing, which promotes the formation of negatively charged groups in the protein structure and consequently shifts the pI toward lower pH values.

**Figure 3:**
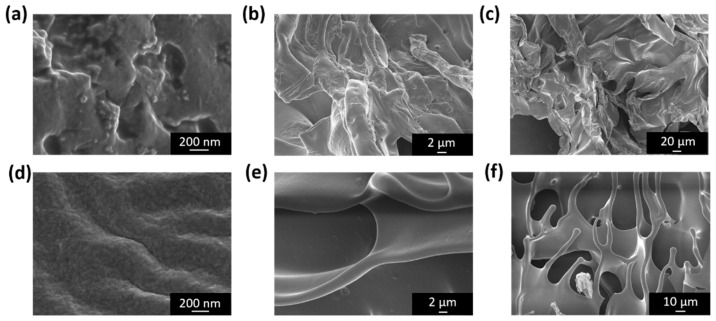
SEM images acquired using an InLens detector at EHT = 3 kV, with a contrast of 25.5% and brightness of 51% in all cases. Micrographs of gelatin samples at different magnifications are shown: (**a**,**d**) 100,000×, (**b**,**e**) 5000×, and (**c**,**f**) 500×, where (**a**–**c**) correspond to HGE gelatin and (**d**–**f**) correspond to UGE gelatin.

To investigate the surface and internal structure of the two gelatin samples, scanning electron microscopy (SEM) analyses were performed using different detectors. In all cases, the samples were freeze-dried and transversely sectioned prior to imaging to allow observation of their internal structure. First, the In-Lens detector, located very close to the objective lens, was employed to collect electron signals immediately after their interaction with the sample. This detector primarily captures low-energy secondary electrons (SEs), providing detailed information on the surface morphology of the material (Figure 3).

From these images, subtle differences in surface morphology between the samples can be observed. The UGE samples (Figure 3d–f) appear to exhibit a slightly smoother and a more homogeneous surface, whereas the HGE sample (Figure 3a–c) shows a somewhat more irregular morphology with the presence of small aggregates. However, these differences are relatively subtle and do not indicate the presence of clearly defined lamellar structures. These morphological variations may be associated with differences in collagen composition and relative abundance between the samples, as suggested by the proteomic analysis (Tables S1 and S2). Such variations could influence the organization of collagen-derived protein aggregates and potentially affect the physicochemical behavior of the material.

**Figure 4:**
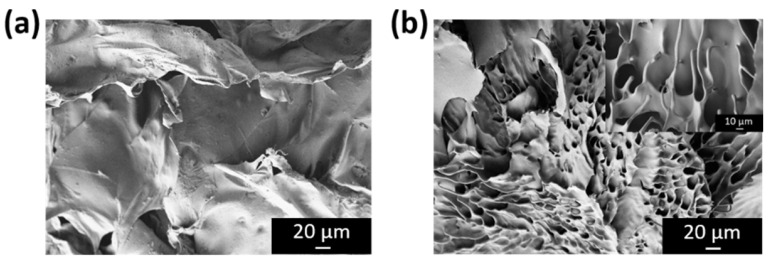
SEM images acquired using an SE2 detector at EHT = 3 kV, with a contrast of 25.5% and brightness of 51% in all cases. Micrographs of gelatin; (**a**) HGE and (**b**) UGE, both at a magnification of 500×, are shown.

Complementarily, images were acquired using an SE2 detector (Figure 4), which is a lateral detector designed to collect secondary electrons emitted radially from the sample surface. Unlike the in-lens detector, the SE2 detector captures a combination of primary secondary electrons (SE1), generated directly by the interaction of the electron beam with the surface, and SE2-type secondary electrons, produced by interactions involving backscattered electrons. This enables enhanced contrast of surface relief and height variations in the sample topography.

In these cross-sectional images, the UGE sample (Figure 4b) exhibited a porous internal structure, with pore sizes ranging from approximately 8 to 16 µm and organized in a thread-like morphology. In contrast, this porous structure was not observed in the HGE sample (Figure 4a), which displayed a more compact internal structure. The formation of this porous network in the UGE sample can be attributed to the effects of ultrasound pretreatment. Acoustic cavitation generates localized shear forces and microjets that disrupt non-covalent interactions, such as hydrogen bonding and van der Waals forces, within the gelatin matrix [4,50,51]. These effects promote structural rearrangements and the formation of a more open and less compact network [25,38]. During subsequent freeze-drying, these structural modifications are preserved, resulting in the porous architecture observed in the SEM images. This increased porosity could be advantageous for the application of collagen in the design of scaffold-type nanocomposites, as it may promote greater colloidal stability, in agreement with the results obtained from zeta potential measurements.

### 3.3. Proximate Composition of the Extracted Gelatins

The proximate composition of the two extracted gelatin samples was determined to obtain additional information on their physicochemical characteristics and overall purity. The moisture content of the HGE sample was 11.65 ± 1.37%, whereas the UGE sample showed a value of 10.91 ± 0.87%. These results are consistent with the mass loss observed in the TGA results (discussed in Section 3.5) within the temperature range of 50–130 °C, which is mainly associated with water evaporation. Regarding mineral residues, the ash content was 1.98 ± 0.04% for the HGE sample and 2.28 ± 0.25% for the UGE sample. Likewise, the total fat contents were 0.87 ± 0.28% and 0.44 ± 0.25%, respectively. These values fall within the ranges reported in previous studies on the proximate composition of gelatin from Nile tilapia scales, where ash contents between 1.19 and 3.18% and fat contents between 0.84 and 1.18% have been described [52]. Although slight differences in mean values observed, no statistically significant differences were found between HGE and UGE samples for any of the evaluated parameters (*p* > 0.05), indicating a comparable proximate composition and similar levels of residual mineral and lipid impurities.

### 3.4. FTIR Characterization of Gelatin Samples

**Figure 5:**
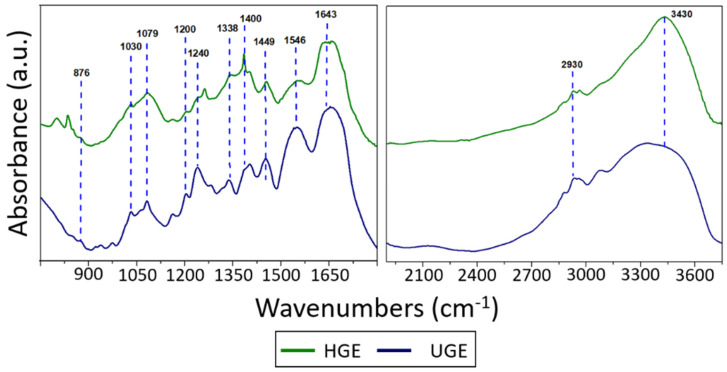
FTIR spectra of gelatin extracted from *Oreochromis niloticus* scales. For clarity, the spectrum is presented in two regions to better highlight the characteristic amide bands of gelatin.

The FTIR spectra of gelatins obtained from Nile tilapia scales, with and without pretreatment, are shown in Figure 5. Both samples exhibit characteristic absorption bands associated with collagen-derived proteins, indicating that the extraction procedure does not significantly modify the fundamental chemical structure of gelatin [53]. In the amide III region (876–1240 cm^−1^), characteristic peaks were observed at 876 cm^−1^, attributed to skeletal stretching vibrations; at 1079 cm^−1^, corresponding to C-O stretching; and at 1240 cm^−1^, assigned to N-H bending coupled with C-N stretching vibrations. These bands are commonly associated with the conformational organization of collagen-derived proteins and reflect the integrity of the peptide backbone [54,55]. Additional minor bands were also observed around 1030 and 1200 cm^−1^. The band near 1030 cm^−1^ can be attributed to C-O or C-O-C stretching vibrations associated with carbohydrate residues or side groups present in collagen, while the band around 1200 cm^−1^ corresponds to contributions within the amide III region related to C-N stretching and N-H deformation of the peptide backbone [56,57].

The amide II band was identified in the range of 1338–1546 cm^−1^, with contributions from CH_2_ wagging and bending vibrations, as well as N-H bending coupled with C-N stretching, confirming the presence of peptide bonds [54,55]. Within this region, a band observed between 1380 and 1400 cm^−1^ can be attributed to CH_2_/CH_3_ bending vibrations associated with amino acid side chains in collagen-derived proteins [58]. The amide I band, located at 1643 cm^−1^ and dominated by C=O stretching vibrations of the peptide bond, is particularly relevant because it is sensitive to protein conformation and commonly used to evaluate structural changes in collagen and gelatin systems [54]. Additionally, the amide B band at 2930 cm^−1^, associated with asymmetric CH_2_ stretching [59], and the amide A band at 3430 cm^−1^, related to N-H stretching coupled with hydrogen bonding interactions, were also observed. These bands reflect typical intermolecular interactions present in collagen-based proteins and indicate that the structural features of gelatin were preserved after the extraction process [60]. The positions of the amide I, II, and III bands observed in the present study are consistent with those commonly reported in the literature for collagen-derived proteins and bovine gelatin, with these bands typically appearing in the ranges of 1300–1400 cm^−1^, 1500–1600 cm^−1^, and 1600–1700 cm^−1^, respectively [61]. These vibrational modes are associated with C=O stretching, N-H bending, and C-N stretching of the polypeptide backbone, which constitute the characteristic infrared signature of gelatin and other protein-based materials [62]. Therefore, these results suggest that ultrasound pretreatment did not significantly modify the fundamental chemical structure of gelatin, as evidenced by the similarity of the spectral features observed for the HGE and UGE samples, although it may influence other physicochemical properties, such as molecular organization or aggregation behavior.

### 3.5. Thermogravimetric Analysis

**Figure 6:**
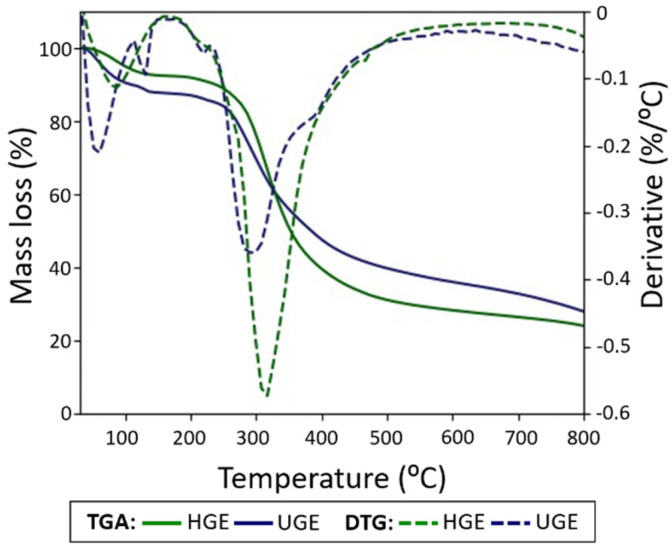
TGA and DTG curves of gelatin samples (HGE and UGE) extracted from Nile tilapia scales.

Thermogravimetric analysis (TGA) curves and their corresponding derivatives (DTG) profiles for the gelatin samples are presented in Figure 6. Both samples exhibited thermal stability within the temperature range of 30–230 °C. In the HGE sample, two thermal events were observed with maxima at approximately 58 and 129 °C, which can be attributed to the loss of free and bound water, respectively. These processes accounted for a combined mass loss of approximately 12% in the temperature range between 30 and 160 °C. The main thermal degradation stage occurred at approximately 293 °C, corresponding to the maximum rate of mass loss and leading to an overall mass reduction of about 38% upon heating up to 500 °C. In contrast, the ultrasound-pretreated sample (UGE) exhibited a single mass loss event at approximately 83 °C, associated with the removal of absorbed water, corresponding to a mass loss of about 9% within the same temperature interval. Additionally, a more pronounced thermal degradation event was observed for UGE, with a maximum at approximately 313 °C, resulting in a total mass reduction of approximately 44% at 500 °C.

The high-intensity peaks identified in the DTG curves of both samples within the temperature range of 200–500 °C can be associated with the thermal decomposition of amino acids present in the gelatin, including the rupture of peptide bonds and hydrogen bonds, particularly within the 240–300 °C range [61]. Although the HGE sample exhibited a higher Mw (212.3 ± 11.8 kDa) than the UGE sample (170.9 ± 13.2 kDa), the latter showed a slightly higher degradation temperature. This behavior suggests that thermal stability was not determined solely by Mw but may also be influenced by changes in intermolecular interactions resulting from ultrasound pretreatment.

Ultrasound generates acoustic cavitation phenomena that produce localized shear forces capable of partially fragmenting collagen chains and modifying their intermolecular interactions [4,63]. These structural alterations may lead to local rearrangements of proteins fragments and changes in aggregation behavior, which can influence the thermal degradation profile of gelatin [64,65]. Similar effects of ultrasound on the structural and thermal properties of collagen-based materials have been reported in previous studies, where cavitation-induced modifications affected protein aggregation, solubility, and thermal stability [4,65,66].

### 3.6. Differential Scanning Calorimetry

**Figure 7:**
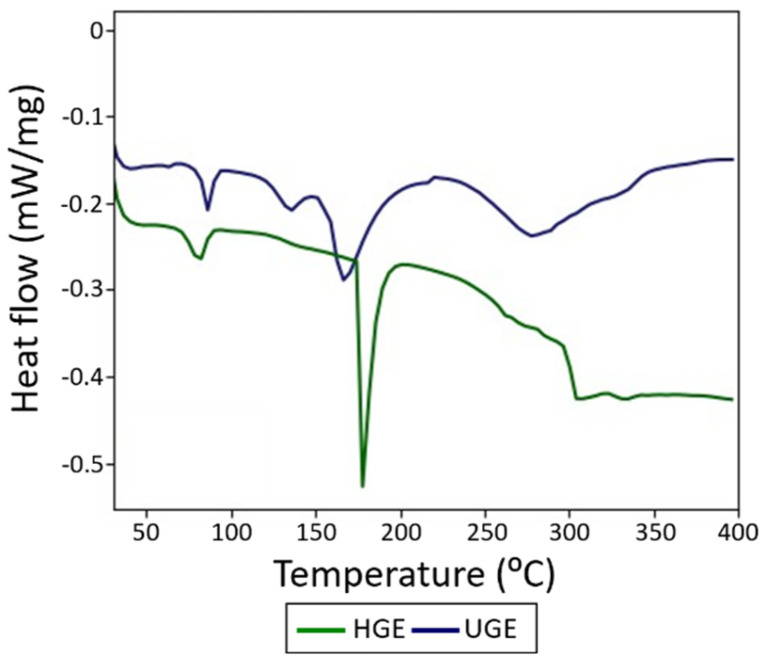
DSC thermograms of gelatin samples extracted from Nile tilapia scales.

The DSC thermograms of both gelatin samples exhibit endothermic activity throughout the analyzed temperature range, with several thermal events associated with structural transitions and bond rupture (Figure 7). For the HGE sample, endothermic peaks are observed at 63.1 °C, 85.9 °C, and 134.6 °C, followed by a broader transition between 150 and 280 °C, with maxima at 167.1 °C and 278.7 °C, after which the signal stabilizes around 300 °C. In contrast, the UGE sample shows a change in slope at 59.2 °C, an endothermic peak at 80.2 °C, and an intense endothermic event in the 180–200 °C range, with a minimum at 177.0 °C, followed by a small peak at 262.0 °C and stabilization of the curve around 303.6 °C. The thermal events observed around 60–65 °C in both samples can be associated with the glass transition temperature (Tg), in agreement with values reported in the literature (23–75 °C) [67]. Additionally, the peak near 80 °C is related to the loss of free moisture, while the peak at 134.6 °C observed in HGE may be influenced by the presence of bound water.

Finally, the endothermic peaks in the 150–220 °C range suggest protein degradation processes, consistent with the mass loss behavior observed in TGA analyses. The broader transitions observed for the HGE sample indicate a more heterogeneous distribution of gelatin chains with different Mw. In contrast, the sharper transitions observed in the UGE sample may reflect a narrower distribution of molecular fragments generated during ultrasound treatment rather than an increase in structural organization. Ultrasound-induced cavitation can promote partial fragmentation of collagen chains and modify intermolecular interactions, which may influence the thermal response of gelatin systems [68].

### 3.7. Gelation Behavior and Water Holding Capacity of the Extracted Gelatins

The gelation ability of gelatin solutions was evaluated through macroscopic observations before and after refrigeration at 4 °C for 1 h. These conditions were selected based on previously reported studies indicating that gelation solutions typically form gels at low temperatures within short time intervals, generally between 10 and 20 min, ensuring sufficient time for network formation [32,69].

Bovine gelatin exhibited gel formation after cooling; however, incomplete dissolution was observed prior to refrigeration, as evidenced by the presence of undissolved aggregates, suggesting limited solubility under the evaluated conditions. In contrast, gelatin solutions (HGE and UGE) showed complete dissolution before cooling, indicating higher solubility compared to bovine gelatin.

After refrigeration, the HGE solutions formed stable gels at both 10 and 5% (*w*/*w*) concentrations, demonstrating their ability to establish a three-dimensional network upon temperature reduction. Conversely, the UGE solutions did not exhibit gelation at any of the concentrations evaluated, despite their complete initial dissolution. Moreover, the UGE samples displayed higher turbidity than the HGE samples, which may be associated with the presence of lower-molecular-weight peptide fragments, and changes in aggregation and hydrophobic behavior induced by ultrasound pretreatment (Figure S3).

To evaluate whether the UGE sample required a longer time to form a gel, additional observations were performed after extended storage at 4 °C. The samples were monitored after 24, 48, and 72 h, and gel formation was not observed at any time point. However, a slight increase in viscosity was detected during storage, suggesting partial structuring of the solution without the formation of a stable gel network (Figure S4). This behavior may be related to structural modifications induced by ultrasound pretreatment. Ultrasound-induced cavitation can promote partial fragmentation of collagen chains and alter molecular weight distribution, which may reduce the ability of gelatin molecules to establish the intermolecular interactions necessary for the formation of a continuous gel network. Similar effects have been reported in previous studies on ultrasound-treated collagen and gelatin systems, where changes in protein structure and aggregation behavior influenced gelation properties [70,71].

Furthermore, the water holding capacity (WHC) of the samples revealed marked differences between the two gelatins. HGE exhibited a significantly higher WHC (430.95 ± 31.8%) compared with UGE (50.66 ± 9.7%). Higher WHC values are generally associated with greater hydroxyproline content and higher molecular weight distributions, which enhance the ability of gelatin molecules to retain water within a protein network [72,73]. In this context, the markedly lower WHC observed for UGE may be attributed to structural modifications induced by ultrasound pretreatment. As discussed above, ultrasound can promote partial fragmentation of collagen chains and alter the molecular weight distribution, thereby weakening the intermolecular interactions required for the formation of a stable gel network. Consequently, the reduced water retention capacity of UGE is consistent with its poor gel-forming ability and the absence of a stable gel structure after cooling. These results are also in agreement with the higher abundance of α1 chains from fibrillar collagens previously identified in the HGE sample compared to the UGE sample [73], which may contribute to the improved network formation and water retention observed in the HGE sample.

### 3.8. Protein Content and Peptide Characterization

Initially, the protein content of both gelatin samples was determined using a calibration curve generated from bovine gelatin as the reference standard. The results indicate that both the HGE sample and the UGE sample, previously subjected to sonication, exhibited high protein contents of 86.7 ± 12.3% and 85.8 ± 13.5%, respectively, with no significant differences observed between the two samples (*p* > 0.05). These values are consistent with previous studies reporting protein contents in gelatin within the range of 85–92% [4,74].

**Figure 8:**
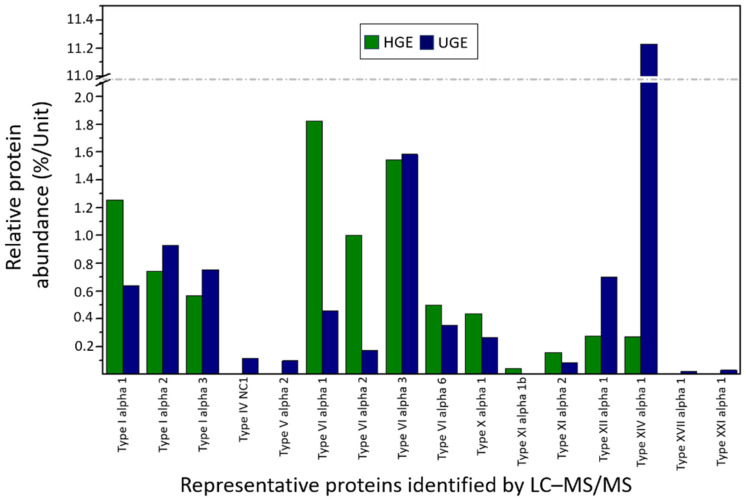
Relative protein abundance (%/Unit) of representative proteins identified in HGE and UGE gelatin samples based on LC–MS/MS proteomic analysis. Protein abundance was estimated using the emPAI index. The horizontal dashed line indicates a break in the y-axis scale.

Furthermore, the peptide composition of both gelatin samples obtained from *Oreochromis niloticus* scales was characterized through LC–MS/MS analysis. In the HGE sample, a total of 661 peptides corresponding to 96 protein types were identified. Among these proteins, 11 showed relative abundances between 0.5 and 1%, while 9 presented abundances higher than 1%, together accounting for 88.1% of the total protein content of the sample (Table S2). A specific analysis of collagen revealed the presence of 12 collagen types, representing 7.51% of the total proteins detected. Figure 8 presents the relative abundance of selected representative proteins identified in the gelatin samples. These proteins include several collagen types (e.g., types I, VI, XI, and XIV) known to play an important role in collagen fibril organization and structural stability [73,75]. In addition, other structural proteins, such as keratins and cytoskeletal proteins, were detected, reflecting the complex protein composition derived from fish scale tissues. The presence and relative abundance of these proteins may influence the structural and physicochemical properties of the extracted gelatin.

In contrast, the UGE sample yielded 2362 peptides corresponding to 337 proteins. Of these, 30 proteins exhibited relative abundances higher than 1%, representing 90.7% of the total protein content of the sample (Table S2). The analysis of collagen proteins revealed the presence of 15 collagen types, which represented 1.76% of the total identified proteins in this sample (Figure 8, Table S3).

To estimate the relative abundance of proteins, the exponentially modified Protein Abundance Index (emPAI) was used as a semi-quantitative indicator of protein abundance. This approach is based on the ratio between the number of observed peptides and the number of theoretically observable peptides for each protein [76]. Therefore, the values presented in Figure 8 correspond to the relative protein abundance estimated from emPAI values. Using this approach, collagen accounted for 8.62% of the total protein abundance in the HGE sample and 17.44% in the UGE sample. These results suggest that, although collagen-related proteins represented a smaller proportion of the total number of identified proteins in UGE, their relative abundance was higher in this sample. These observations indicate that ultrasound pretreatment may enhance the extraction and solubilization of collagen from *Oreochromis niloticus* scales. Finally, the proteomic datasets generated for both gelatin samples have been deposited in the PRIDE repository under accession number PXD071911.

## 4. Conclusions

In this study, type B gelatin was successfully extracted from *Oreochromis niloticus* scales under hydrothermal conditions, confirming fish scales as a suitable and sustainable source of this biopolymer. Ultrasound-assisted pretreatment slightly increased gelatin yield and promoted collagen solubilization, as supported by proteomic analysis, which showed a higher relative abundance of collagen-related proteins in the UGE samples. However, ultrasound treatment also induced structural modifications that significantly influenced the functional behavior of the extracted gelatin. In particular, the ultrasound-treated sample showed a reduction in Mw, suggesting partial fragmentation of collagen chains caused by cavitation effects. These structural changes were reflected in the functional properties of the material, as the UGE samples exhibited limited gel-forming capacity and significantly lower water holding capacity compared with the HGE sample.

By contrast, gelatin extracted without ultrasound preserved higher-Mw fractions and demonstrated effective gelation at both 5 and 10% (*w*/*w*), highlighting the importance of maintaining collagen chain integrity for the formation of stable gel networks. Thermal and spectroscopic analyses further confirmed that the fundamental chemical structure of collagen was preserved in both samples, although differences in thermal behavior reflected variations in molecular organization.

Overall, these results suggest that ultrasound-assisted extraction involves a balance between enhanced collagen recovery and structural alterations that may compromise gelation performance. Therefore, extraction conditions should be carefully evaluated according to the intended application, particularly when balancing collagen availability with the structural integrity required for gel formation and water retention, which are very important factors for the development of collagen-based materials in pharmaceutical and biomedical applications.

## Data Availability

The mass spectrometry proteomics data corresponding to the characterization of both gelatin samples have been deposited to the ProteomeXchange Consortium via the PRIDEpartner repository with the dataset identifier PXD071911.

## Supplementary Materials

The following supporting information can be downloaded at https://www.mdpi.com/article/10.3390/pharmaceutics18040463/s1, Figure S1: Calibration plots of *KC/R_Ѳ_* as a function of concentration used for the estimation of the MW of fish gelatin samples obtained through hydro-extraction (HGE) and ultrasound-assisted extraction (UGE) using SLS. Figure S2. Determination of the isoelectric point (pI) of HGE and UGE gelatin samples. (a) Titration curves obtained through controlled addition of NaOH (0.1 M) to gelatin solutions previously dissolved in HCl (0.25 M). (b) Second derivative of the titration curves used to identify the inflection point corresponding to the pI. The calculated pI values were 4.41 for HGE and 4.90 for UGE. Figure S3: Gelation capacity of type B gelatin samples (HGE and UGE) compared with bovine gelatin used as the reference. Panels (a,d) show bovine gelatin before and after refrigeration at 4 °C; (b,e) show the HGE sample; and (c,f) show the UGE sample under the same conditions. Refrigeration was carried out at 4 °C for 1 h. Figure S4. Gelation behavior of ultrasound-treated gelatin samples (UGE) during refrigerated storage at 4 °C. (a) After 1 h and (b) after 72 h. Table S1. Hydrodynamic diameter and polydispersity index (PDI) of gelatin samples as a function of dispersion method and temperature prior to DLS measurements. Table S2: Proteomic characterization of hydro-extracted (HGE) and ultrasound-assisted extracted (UGE) gelatin samples based on identified peptides. Table S3: Collagen abundance in hydro-extracted (HGE) and ultrasound-assisted extracted (UGE) samples estimated by the exponentially modified protein abundance index (emPAI).
