# Supplementary material for: Physicochemical and Functional Properties of Type B Gelatin Obtained from Nile Tilapia (Oreochromis niloticus) Scales Using Hydro-Extraction: Effect of Ultrasound Pretreatment
Source: Pharmaceutics. 2026 Apr 9;18(4):463. doi: 10.3390/pharmaceutics18040463 (PMC13119067; doi:10.3390/pharmaceutics18040463)
Supplement: Supplementary file 1 [file pharmaceutics-18-00463-s001.zip › pharmaceutics-4151407-supplementary.pdf]

Physicochemical and functional properties of type B gelatin obtained from Nile tilapia (*Oreochromis niloticus*) scales by hydro-extraction: effect of ultrasound pretreatment

Kelly Triana-Jiménez, Carlos Alonso, Milena A. Vega, Pablo Juanes-Velasco, Iván Meneses-Rivera and Mario Velásquez-Lozano

Supplementary Material

Molecular weight determination by SLS

The molecular weight (MW) analysis of the two gelatin samples (HGE and UGE) was carried out using static light scattering (SLS). For this purpose, ten solutions of each gelatin sample were prepared and dissolved in phosphate-buffered saline (PBS, pH 7.4) over a concentration range of 0.01 to 0.1 g/mL, using toluene as the Rayleigh ratio standard. The gelatin solutions were subsequently introduced into the instrument at the corresponding concentrations, and the scattering signals were recorded. From these measurements, the software generated a first-order linear relationship, in which the intercept corresponds to the inverse of the weight-average molecular weight (1/MW), while the slope is associated with the second virial coefficient (A_2), according to Equation S1 [1]:

$$\frac{KC}{R_\theta} = \left(\frac{1}{MW} + 2A_2C\right)P(\theta) \qquad \textbf{Ec.S1}$$

where C is the polymer concentration in solution, R_θ is the Rayleigh ratio, defined as the intensity of scattered light at an angle θ, MW is the weight-average molecular weight of the polymer, A_2 is the second virial coefficient describing polymer–solvent interactions, and $P(\theta)$ is the angular form factor related to the size and conformation of the macromolecule, which for large polymers depends on the radius of gyration (Rg).

The optical constant of the system (K) was calculated according to Equation S2:

$$K = \frac{4\pi^2 n_0^2}{N_A \lambda^4}\left(\frac{d_n}{dC}\right)^2 \qquad \textbf{Ec.S2}$$

where n_0 is the refractive index of the solvent, λ is the wavelength of the incident light, dn/dC is the refractive index increment, and N_A is Avogadro´s number.

Finally, Figure S1 shows the calibration plot used to determine the MW of the two gelatin samples analyzed in this study.

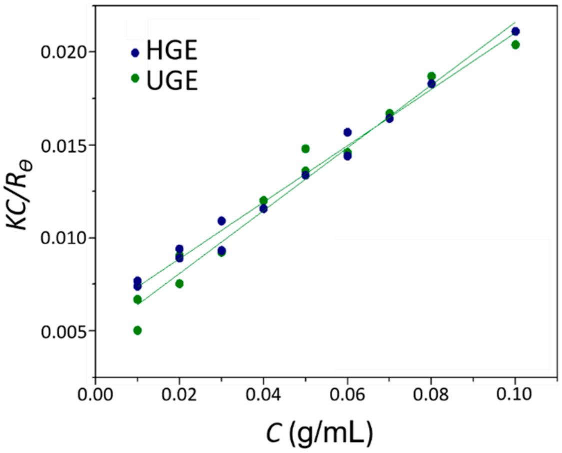

$$\frac{KC}{R_{\theta\ (HGE)}} = 0.16909C + 0.00471 \qquad \frac{KC}{R_{\theta\ (UGE)}} = 0.15201C + 0.00585$$

Figure S1. Calibration plots of KC/R_θ as a function of concentration used for the estimation of the MW of fish gelatin samples obtained by hydro-extraction (HGE) and ultrasound-assisted extraction (UGE) using SLS.

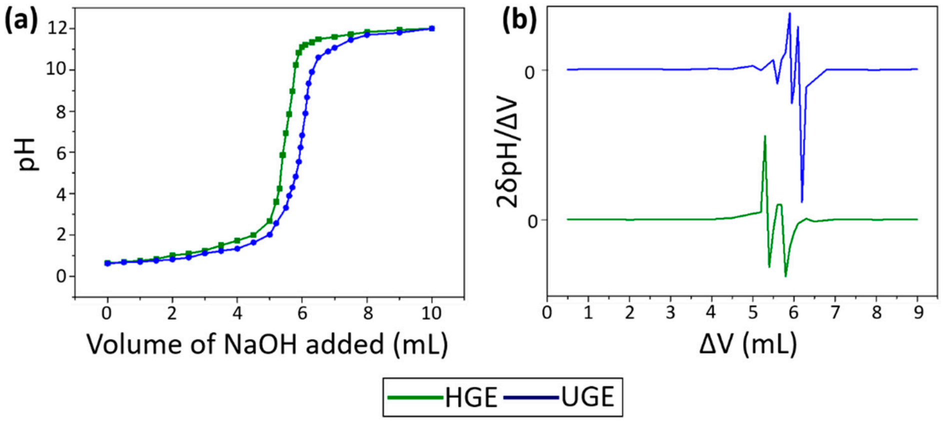

Figure S2. Determination of the isoelectric point (pI) of HGE and UGE gelatin samples. (a) Titration curves obtained by controlled addition of NaOH (0.1 M) to gelatin solutions previously dissolved in HCl (0.25 M). (b) Second derivative of the titration curves used to identify the inflection point corresponding to the pI. The calculated pI values were 4.41 for HGE and 4.90 for UGE.

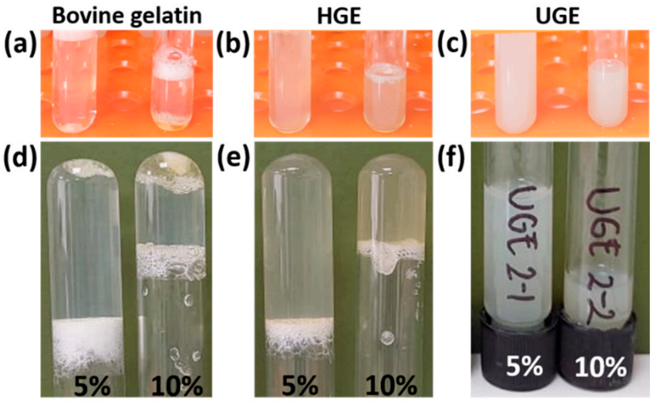

Figure S3. Gelation capacity of type B gelatin samples (HGE and UGE) compared with bovine gelatin used as the reference. Panels a and d show bovine gelatin before and after refrigeration at 4 °C; b and e show the HGE sample; and c and f show the UGE sample under the same conditions. Refrigeration was carried out at 4 °C for 1 h.

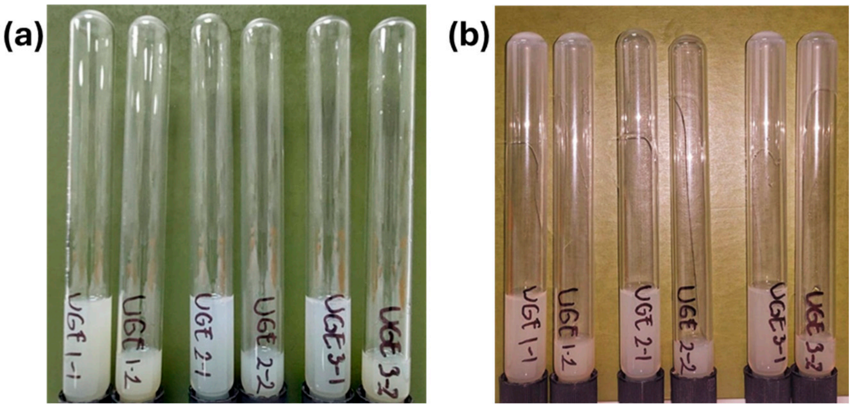

Figure S4. Gelation behavior of ultrasound-treated gelatin samples (UGE) during refrigerated storage at 4 °C. (a) After 1 h and (b) after 72 h.

Table S1. Hydrodynamic diameter and polydispersity index (PDI) of gelatin samples as a function of dispersion method and temperature prior to DLS measurements.

Sample	Treatment	Hydrodynamic diameter (nm)	PDI
**HGE-20 °C**	Control	313.87 ± 39.10	0.470
	Vortex	304.70 ± 29.26	0.530
	Sonication	269.45 ± 12.09	0.516
**HGE-50 °C**	Control	338.53 ± 28.32	0.660
	Vortex	236.68 ± 13.20	0.810
	Sonication	281.4 ± 20.43	0.554
**UGE-20 °C**	Control	--	--
	Vortex	--	--
	Sonication	367.3 ± 26.3	0.516
**UGE-50 °C**	Control	--	--
	Vortex	--	--
	Sonication	378.1 ± 16.97	0.554

Table S2. Proteomic characterization of hydro-extracted (HGE) and ultrasound-assisted extracted (UGE) gelatin samples based on identified peptides.

Protein Description	HGE (%)	UGE (%)
**Actin, Beta**	--	2.400
**AHNAK nucleoprotein**	0.811	1.172
**Alpha-1-antitrypsin homolog**	--	1.244
**Annexin(2Mut)**	--	2.738
**Apolipoprotein A-Ib**	--	2.195
**Cofilin 1 (non-muscle)**	--	1.507
**Collagen type XIV alpha 1**	--	1.132
**Decorin**	8.821	1.662
**Fibrinogen beta chain**	--	1.108
**Fibrinogen gamma chain**	--	1.879
**Hemoglobin subunit alpha-B**	0.761	1.383
**Hemoglobin subunit epsilon 1(3Mut)**	20.022	-
**Hemoglobin subunit epsilon 1(7Mut)**	--	23.511
**Histone H2A(2Mut)**	14.497	3.660
**Histone H4**	12.560	1.350
**Ictacalcin**	0.554	-
**IF rod domain-containing protein (2Mut)**	1.249	-
**IF rod domain-containing protein(5Mut)**	--	11.434
**Keratin 15**	--	2.346
**Keratin 18a**	--	2.202
**Keratin 18b**	--	2.093
**Keratin 5**	11.371	2.86
**Keratin 8**	--	1.656
**Keratin 9**	--	2.072
**Keratin, type II cytoskeletal 8**	--	1.334
**LOC100711401**	0.765	1.296
**Lumican**	0.642	-
**NME/NM23**	0.581	
**Protein S100-A16**	--	2.065
**Si:ch211-156l18.7**	0.750	3.224
**Thioredoxin**	--	1.323

**Tropomyosin 4a**	--	1.383
**Tropomyosin 4b (2Mut)**	--	2.598
**Type I alpha 1**	0.652	0.065
**Type I alpha 1(2Mut)**	1.093	-
**Type I alpha 2**	0.645	0.094
**Type I alpha 3**	0.493	0.076
**Type IV NC1 (2Mut)**	--	0.011
**Type V alpha 2**	--	0.01
**Type VI alpha 1**	--	0.046
**Collagen, type VI, alpha 1(2Mut)**	1.589	-
**Type VI alpha 2**	0.724	0.017
**Type VI alpha 1(2Mut)**	1.589	-
**Type VI alpha 2(3Mut)**	0.872	-
**Collagen type VI alpha 3**	0.89	-
**Type VI alpha 3 (2Mut)**	1.343	0.16
**Type VI alpha 6 (2Mut)**	0.434	0.036
**Type X alpha 1**	--	0.027
**Type X, alpha 1(2Mut)**	0.382	-
**Type XI alpha 2**	--	0.009
**Type XI alpha 2(2Mut)**	0.137	-
**Type XI, alpha 1b**	0.0389	-
**Type XII alpha 1 (2Mut)**	0.242	0.071
**Type XIV alpha 1**	--	1,132
**Type XIV alpha 1(2Mut)**	0.235	-
**Type XVII alfa1**	--	0.002
**Type XXII alpha 1**	--	0.003
**Ubiquitin B**	8.984	1.041
**VWFA domain-containing protein (2Mut)**	1.276*	4.819

Table S3. Collagen abundance in hydro-extracted (HGE) and ultrasound-assisted extracted (UGE) samples estimated by the exponentially modified protein abundance index (emPAI).

EmPAI Score	HGE	UGE
**Type I alpha 1**	1.2564	0.6406
**Type I alpha 2**	0.7418	0.9275
**type I alpha 3**	0.5668	0.7539
**Type IV NC1**	--	0.1138
**Type V alpha 2**	--	0.099
**Type VI alpha 1**	1.826	0.4588
**Type VI alpha 2**	1.0026	0.1722
**Type VI alpha 3**	1.5435	1.584
**Type VI alpha 6**	0.4991	0.3541
**Type X, alpha 1**	0.4390	0.2647
**Type XI, alpha 1b**	0.0447	--
**Type XI alpha 2(2Mut)**	0.1579	0.0864
**Type XII alpha 1(2Mut)**	0.2788	0.701
**Type XIV alpha 1(2Mut)**	0.270	11.232
**Type XVII alfa1**	--	0.0209
**Type XXII alpha 1**	--	0.0304

References

1. Rodriguez-Loya, J.; Lerma, M.; Gardea-Torresdey, J.L. Dynamic light scattering and its application to control nanoparticle aggregation in colloidal systems: a review. *Micromachines* **2023,** *15, 24.* https://doi.org/10.3390/mi15010024. Reference [30] in the main text.
